# Management of Acute Saddle Pulmonary Embolism in Pregnancy Following Fetal Surgery

**DOI:** 10.7759/cureus.54607

**Published:** 2024-02-21

**Authors:** Patrick J Connell, Leonardo A Marquez Roa, Jorge Araujo-Duran, Monica Cheriyan, Sabry Ayad

**Affiliations:** 1 Anesthesiology and Perioperative Medicine, Cleveland Clinic, Cleveland, USA; 2 Outcomes Research, Cleveland Clinic, Cleveland, USA; 3 Outcomes Research, Cleveland Clinic Fairview Hospital, Anesthesiology Institute, Cleveland, USA; 4 Obstetric Anesthesiology, Cleveland Clinic, Cleveland, USA; 5 Anesthesiology, Cleveland Clinic, Cleveland, USA; 6 Anesthesiology, Cleveland Clinic Fairview Hospital, Cleveland, USA

**Keywords:** bleeding in pregnancy, therapeutic anticoagulation, anticoagulation, critical care obstetrics, intravenous thrombolytic therapy, ivc thrombus, high-risk pregnancy, massive pulmonary embolism, pulmonary embolism, fetal surgery

## Abstract

A 33-year-old gravidity three parity three (G3P3) woman at 34 weeks of pregnancy underwent fetal surgery to repair an open lumbosacral myelomeningocele at 22 weeks gestation and experienced preterm premature rupture of membranes as a result. She developed a saddle pulmonary embolus with signs of right heart strain while on prolonged bed rest. She was treated emergently with aspiration thrombectomy and suprarenal inferior vena cava (IVC) filter placement, followed by an uncomplicated cesarean delivery thereafter.

## Introduction

Thromboembolic events are one of the leading causes of death during pregnancy, accounting for 9.2% of all pregnancy-related deaths - an incidence that is 10 times higher than in non-pregnant populations [1]. The physiologic changes in pregnancy increase the likelihood of these events, particularly hypercoagulability and venous stasis. Current guidelines recommend immediate introduction of low-molecular-weight heparin upon confirmation of a thromboembolic event, yet hemorrhage is an ever-present concern in the parturient [2]. When complicating or concerning medical history, such as recent surgery, is included in decision-making, the appropriate care path becomes only more unclear.

The course of her care exemplifies the challenges that may arise during pregnancy as a result of the recent advances in fetal surgery both in exposure to complications from the intervention and a hurdle to determining optimal management strategy in emergencies.

## Case presentation

This patient was a 32-year-old gravidity three parity three (G3P3) female with a past medical history of obesity and remote dilation and curettage (D&C) for incomplete spontaneous abortion. Her pregnancy was complicated by a routine ultrasound finding the fetal presence of open lumbosacral myelomeningocele at L5 with osseous defect and hernia sac and Chiari II malformation characterized by bilateral ventriculomegaly at 20 weeks of pregnancy. Fetal surgery successfully repaired the open neural tube defect under general anesthesia with a lumbar epidural placed for pain control at 22 weeks of pregnancy. Her pregnancy was further complicated by a preterm premature rupture of membranes at 31 weeks, which was treated with latency antibiotics, and betamethasone was given for fetal lung maturity. Activities were restricted to full bedrest while remaining under inpatient care, which was planned for the remainder of her pregnancy with plans for cesarean delivery at 34-35 weeks gestation.

**Figure 1:**
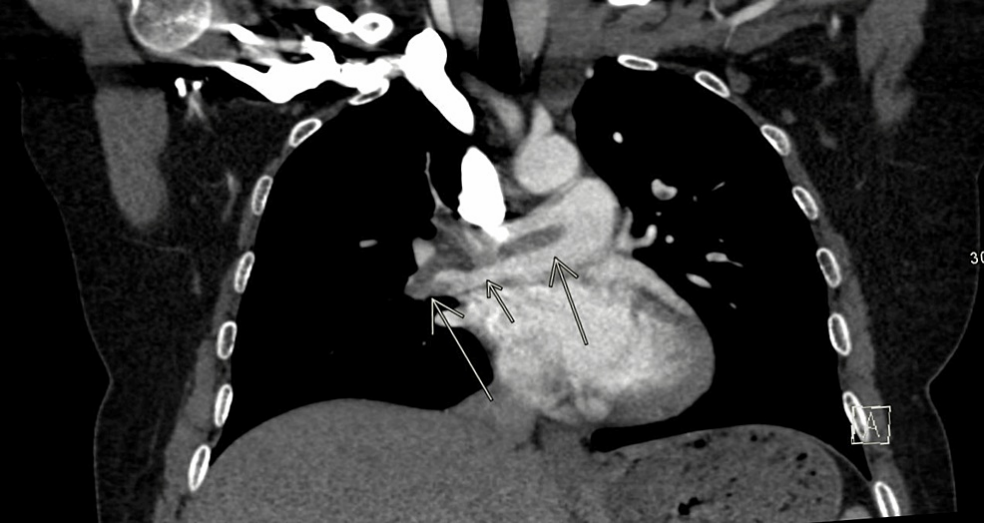
Coronal pulmonary CT angiogram demonstrating the longest in-plane view of the thrombus seen across the pulmonary arteries.

**Figure 2:**
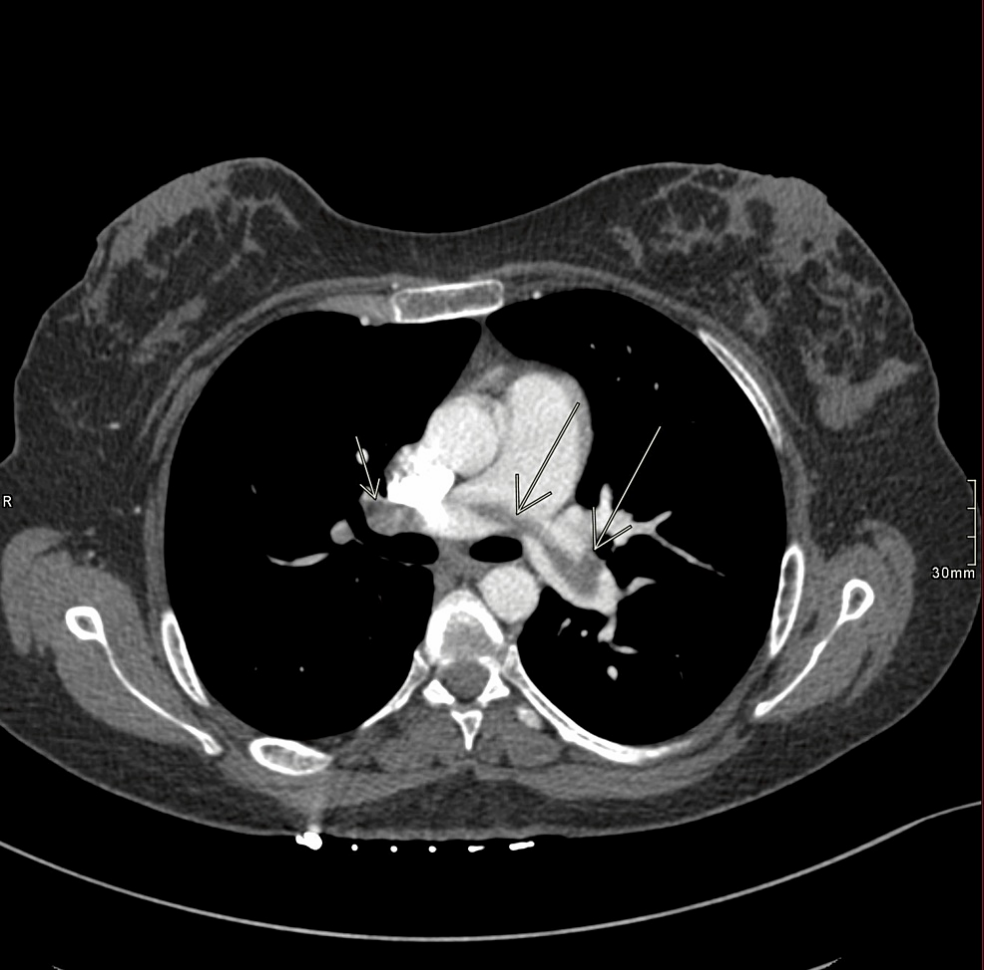
Transverse CT pulmonary angiogram demonstrating the largest transection of the pulmonary thrombus, which measured approximately 0.5 cm wide and spanned over 10 cm from end to end in the main pulmonary arteries before branching into subsegmental arteries (not included in this view).

Her stay was uneventful other than light, intermittent vaginal bleeding until 34 weeks gestation when she was seen at the bedside and noted to have shortness of breath, palpitations, and pleuritic chest pain, which occurred hours after intermittent right upper-medial thigh pain. Oxygen saturations declined to ~87%, and she became tachycardic to 136 BPM and was placed on 15 liters of oxygen via a non-rebreather, which was weaned to 2 L via nasal cannula over the following three hours. Computed tomography (CT) pulmonary angiogram revealed a saddle embolus across the main pulmonary arteries with multiple, bilateral global filling defects, which appeared worse on the right side, and moderate distension of the right heart chambers (Figures 1, 2). The clot appeared to be 0.5 cm wide and spanned over 10 cm from end to end in the main pulmonary arteries before branching into segmental arteries bilaterally. Labs performed included a troponin T peak of 0.119 and a D-dimer of 10,930 ng/mL FEU. The YEARS algorithm, “a diagnostic algorithm that determines the risk of pulmonary embolism (PE) derived from three items in the Wells score,” suggested that anticoagulation was the next best step, and heparin infusion was initiated. She was transferred via a helicopter to the medical intensive care unit (MICU) at a nearby tertiary facility with extracorporeal membrane oxygenation (ECMO) capabilities. Her intermittent vaginal bleeding progressed into active bleeding, raising concerns for placental abruption or uterine rupture, and continuous fetal monitoring was initiated. Despite this bleeding, heparin infusion was continued due to significant clot burden.

A multidisciplinary team including maternal-fetal medicine, interventional radiology, and pulmonary embolism response team (PERT) assessed the potential for maternal and fetal decompensation that would require emergency cesarean delivery. The clot burden was deemed very likely to lead to decompensation during delivery, so she was prepared for aspiration thrombectomy and inferior vena cava (IVC) filter placement with cesarean delivery to follow. Prior to operative intervention, other labs and studies collected included a complete blood panel (CBC) with hemoglobin of 10.4 g/dL (normal range 12.0-16.0 g/dL for women), prothrombin time international normalized ratio (PT/INR) of 0.9 (normal range 0.8 to 1.1), activated partial thromboplastin time (aPTT) of 26.9 seconds (normal range 25 to 35 seconds), and type and screen. Bedside echocardiography was performed prior to surgery, which reconfirmed right heart dilation, but no formal report or quantification of the dysfunction was created. Category I tracings on fetal monitoring were maintained and active bleeding ceased prior to surgery. Transabdominal uterine ultrasound at the bedside did not visualize signs of placental abruption or uterine rupture.

Intraoperative care for this patient was largely negotiated by risk stratification. Monitors included continuous EKG, oxygen saturation, and non-invasive blood pressure per American Society of Anesthesiologists (ASA) standard guidelines. A radial artery invasive blood pressure measurement was established, as well as a central line access in the right internal jugular vein for medication administration and the right femoral vein for completion of the thrombectomy. ECMO was on standby for both operating room events but was not required. The anesthetic plan was monitored anesthesia care with the goal of avoiding hemodynamic instability and exacerbation of right heart dysfunction that may occur during both induction of general anesthesia and thrombectomy. The literature evidences a high chance for decompensation during thrombectomy for parturients, which then requires the introduction of ECMO. She was given versed, propofol bolus, fentanyl, 250 mL 5% albumin, and 1.5 L crystalloid. She remained stable throughout the procedure with blood pressure remaining at 20% of her baseline ~100/65, and the heart rate downtrended from 130 to 90. She remained on heparin infusion throughout the procedure and was then held upon removal of the clot. Pulmonary artery pressure prior to removal was 35/15 mmHg (mean 26) and was 19/8 mmHg (mean 13) post-thrombectomy. There was a blood loss of 300 mL.

She returned to the operating room two hours later for her cesarean delivery, which was performed under spinal anesthesia with IV analgesia (midazolam, ketamine, fentanyl boluses, and propofol infusion). Norepinephrine infusion was used for hemodynamic support, which successfully maintained blood pressures within 20% of baseline, and she was given 250 mL 5% albumin and 2.25 L crystalloid. She was given two units of packed red blood cells and four units of fresh frozen plasma (FFP) during the thrombectomy. Blood loss was 950 mL. Immediately after the delivery, therapeutic heparin infusion was re-initiated, and therapy was monitored with scheduled anti-factor Xa assay, PT/INR, and aPTT. She received an additional three units of packed red blood cells in the subsequent three days, but post-operative hemoglobin measurements continued to downtrend. Labs were ordered to monitor anticoagulation and investigate coagulopathy in the setting of decreasing hemoglobin, and fibrinogen remained unchanged at 429 mg/dL (normal range 200 to 400 mg/dL), aPTT was within the therapeutic range, and platelets remained stable at ~170,000 platelets per microliter (normal range 150,000 to 450,000 platelets per microliter).

On post-operative day 4, she developed progressing abdominal and chest pain. CT of the abdomen and pelvis demonstrated a 15 x 14 x 8 cm rectus sheath hematoma in the anterior pelvis and a small nonocclusive filling defect in the middle hepatic vein that was suspected to be a thrombus. Therapeutic heparin was held with plans for restart in 10-14 days. Aggressive deep vein thrombosis (DVT) prophylaxis and intermittent pneumatic compression (IPC) stockings, thromboembolic deterrent (TED) hose, and adequate hydration were utilized in the meantime. On post-operative day 6, a repeat CT abdomen revealed that her hematoma had stabilized but a new, non-occlusive supra-IVC filter thrombus developed that extended into the right renal vein, causing approximately 50% occlusion. Therapeutic heparin infusion was then restarted, and her hemoglobin level remained stable. She was transitioned to subcutaneous low-molecular-weight heparin (LMWH) after three days and was discharged with plans for transition to warfarin in three weeks and close follow-up with maternal-fetal medicine and vascular surgery.

During outpatient follow-up, echocardiography was repeated, which demonstrated resolution of the right heart dilation seen on CT pulmonary angiogram. Tests for congenital coagulopathy were ordered, including a lupus anticoagulant panel, cardiolipin antibody, beta-2 glycoprotein antibody, and tests for inherited thrombophilia - prothrombin gene, APC resistance, protein C/S, and antithrombin III levels.

## Discussion

Pulmonary embolism is the third most common cardiovascular disease behind coronary artery disease and stroke with an annual incidence of approximately 70 per 100,000, and current data suggest that rates may be much higher due to the presence of a “silent PE” formation in up to 50% of patients with DVT [3-4]. Of note, autopsy reports show that a mere 30-35% of PE are diagnosed before death [5]. Furthermore, venous thromboembolism is more common during and shortly after pregnancy due to increases in coagulation factors I, II, VII, IX, and XII, decreases in protein C, and impaired fibrinolysis with increased plasminogen activator inhibitors (PAI-1 and PAI-2). The presence of venous stasis from a high plasma volume state, requirement for prolonged postpartum bed rest, and high risk for intravascular wall damage increase risk further - in many ways, pregnancy is the “perfect storm” of Virchow’s triad. Risks for an event are only elevated by comorbidities that increase hypercoagulability, such as pre-existing heart disease, obesity, anemia, diabetes, hypertension, and pregnancy complications, such as antepartum hemorrhage, cesarean delivery, and requirement for transfusion [5].

**Figure 3:**
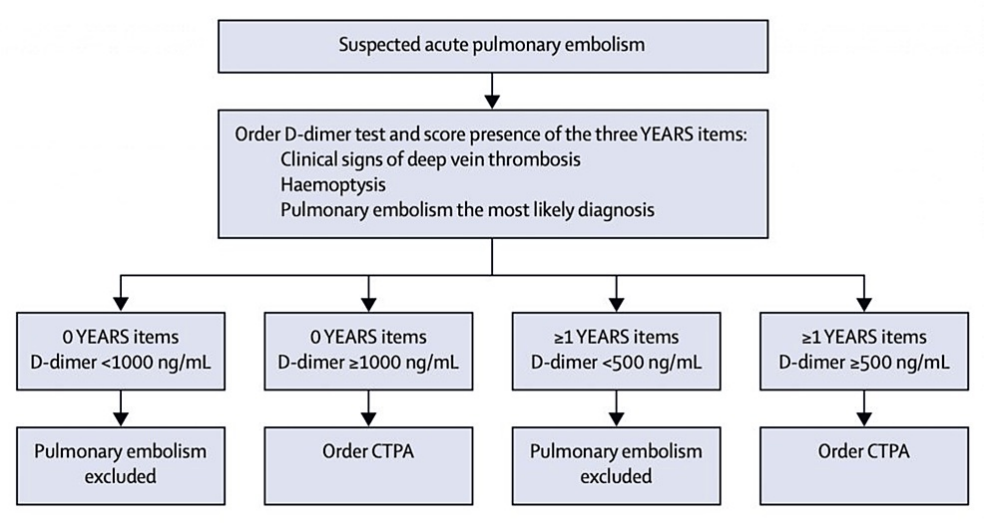
YEARS algorithm CTPA: computed tomography pulmonary angiography

Testing for pulmonary embolism is outlined by the YEARS algorithm, which aims to correlate the presence of clinical signs with D-dimer to determine whether CT pulmonary angiography imaging is appropriate (Figure 3). Radiation exposure to a pregnant mother and her fetus is a risk not to be taken lightly, and the YEARS algorithm has been adapted for pregnancy to include preliminary ultrasound imaging for DVT in hopes of avoiding its use to detect abnormalities when present. When found, these guidelines lead to the initiation of anticoagulation therapy immediately, most typically low-molecular-weight-heparin and less often tissue plasminogen agents, such as alteplase [6].

Fetal surgery is a relatively new means of addressing congenital abnormalities where invasive repair during the second trimester outweighs the risks of delivery and neonatal surgery. Although this development in care is revolutionary, the risk for any maternal complication has been reported as 20.9% for open fetal procedures and 6.2% for fetoscopic surgery [7]. Of great concern, serious and life-threatening maternal complications make up 4.5% and 1.7% of these, respectively [8]. Of those categorized as severe complications, placental abruption and uterine rupture were by far the most commonly seen. Both pathologies often present with vaginal bleeding with or without the presence of pain. Other complications that have been reported as a result of fetal surgery include chorioamnionitis and sepsis, pulmonary edema, hemorrhage requiring both transfusion and urgent delivery, and bowel obstruction. There is a direct correlation between the number of fetal interventions and the likelihood of iatrogenic preterm premature rupture of membranes with an incidence as high as 48% after open surgery and 36% follow fetoscopic procedures [6]. This is due to the inability of the gestational sac to fully heal after the intervention [9]. It is reasonable to connect the occurrence of PPROM in this mother to the prior fetal surgery. Due to her PPROM, she required inpatient management of her condition, which included orders for bed rest and activities limited to minimal ambulation and resting in a bedside chair. Intermittent bleeding during this time was a contraindication for subcutaneous heparin administration as a form of DVT prophylaxis [10].

The past medical history of fetal surgery in the setting of confirmed pulmonary embolism creates a landscape for decision-making with little guiding evidence in the literature to rely on. In the presence of active vaginal bleeding, one is risking that there may be a complication that has been masked by other symptoms or otherwise undetected. If thrombolytic therapy is given to treat pulmonary embolism, a placental abruption or uterine rupture has the potential to escalate to an immediate life-threatening event. In either event, urgent/emergent delivery is required. Resolution of the clot burden must then be triaged against delivery of the fetus, and the probability of decompensation during delivery due to an ongoing cardiopulmonary event is far too high to risk. This is reason enough to utilize invasive modalities for intervention including placement of IVC filter and either catheter-directed vs. surgical thrombectomy. Oftentimes, extracorporeal membrane oxygenation is a part of the management plan for therapy, which can be used as sole therapy or bridging to thrombectomy.

This management is the safest effective plan for a mother with a history of fetal surgery and predisposition for complications from anticoagulation. In this patient, catheter-related thrombectomy and IVC filter placement successfully resolved the embolus without longstanding complications on her cardiac or pulmonary function as demonstrated by postoperative echocardiography, and it made successful subsequent cesarean delivery a safe and uncomplicated procedure as well.

Preventative measures must be put in place to decrease the likelihood of thrombotic events in the setting of bleeding in PPROM. Guidelines for more rigorous activity may have minimized the potential for venous stasis and development of DVT, which occurred in this patient who experienced right medial thigh pain hours prior to her shortness of breath and chest pain. There are numerous mechanical prophylaxis strategies that may be utilized, including physiotherapy and exercises, use of graduated compression stockings, foot pumps, and intermittent pneumatic compression devices [11]. Conversely, these activities may be contraindicated in mothers with a history of fetal surgery who have not experienced PPROM as it would increase the risk of it occurring. For those who experience PPROM, more vigorous activity may lead to undesirable progression toward labor despite efforts to delay. Best management must be determined on a patient-by-patient basis using informed clinical judgment. Patient education and awareness play an important role in early venous thromboembolism (VTE) detection as well. As few as 25% of patients interviewed in global surveys were aware that there was an increased risk of VTE events while hospitalized [12]. Early reporting of symptoms can lead to timely intervention and prevent a worse clinical scenario. Tools for the prediction of risk, such as the Padua Risk Assessment Model, have been developed, but their use has yet to be applied to patients in an obstetric setting to a degree that guides the use of mechanical prophylaxis.

## Conclusions

Pregnancy is a state that alters baseline physiological function in a multitude of ways. Many of these changes align Virchow’s triad toward a much greater chance of thromboembolic events. The emergence of interventions, such as fetal surgery, are revolutionary means to improve outcomes for neonates but also carry considerable risks for both the mother and fetus, many of which can be life-threatening.

Current guidelines for therapy for pulmonary embolism in pregnancy primarily rely on thrombolysis, which exposes mothers with a history of fetal surgery to exceptionally high risk. This case demonstrates how this event can be successfully managed with immediate thrombolysis, mechanical thrombectomy, and IVC filter placement and allow for safe delivery of the fetus.
